# Combined nutrition and physical activity education for early adolescents with malnutrition: A systematic review of overweight interventions and the undernutrition evidence gap

**DOI:** 10.1371/journal.pgph.0007280

**Published:** 2026-09-29

**Authors:** Radiet Kidane Alemu, Tefera Belachew, Beyene Wondefrashe, Eugène Rameckers, Kenneth Verboven, Evi Verbecque

**Affiliations:** 1 Department of Nutrition and Dietetics, Faculty of Public Health, Jimma University, Jimma, Ethiopia; 2 REVAL Rehabilitation Research Center, Faculty of Rehabilitation Sciences, Hasselt University, Diepenbeek, Belgium; 3 Department of Rehabilitation Medicine, Maastricht University, Maastricht, the Netherlands; 4 Adelante Pediatric Rehabilitation, Center of Expertise, Hoensbroek, the Netherlands; Qatar University College of Medicine, QATAR

## Abstract

Early adolescence represents a pivotal stage of growth and development, characterized by profound changes in dietary patterns and physical activity behaviors. Yet, despite the well-documented consequences of both undernutrition and overnutrition, this age group remains largely overlooked in low- and middle-income country settings where the double burden of malnutrition is rapidly intensifying. The primary objective of this systematic review is to determine the effect of combined physical activity and nutrition education interventions among early adolescents experiencing either underweight or overweight, living in socio-economically disadvantaged circumstances. PubMed, Web of Science, Scopus, and LILACS Plus collection were systematically searched and last updated on June18^th^ 2026. The methodological quality of the studies was assessed using the revised Cochrane Risk of Bias tool for randomized trials. The Grading of Recommendations, Assessment, Development, and Evaluation approach was used to assess the quality of evidence.Five studies comprising 960 adolescents all classified as overweight or obese met the inclusion criteria; no studies involving underweight adolescents were identified. Risk of bias was generally low to some concerns. Overall, the evidence suggests that combined physical activity and nutrition education interventions may improve anthropometric indicators, dietary behaviors, physical activity levels and sedentary behaviors among overweight and obese early adolescents. However, the overall certainty of evidence was very low primarily because of substantial heterogeneity and the limited number of available studies.Given the very low certainty of evidence, the effectiveness of combined nutrition and physical activity education interventions among overweight and obese early adolescents living in resource-constrained settings remains uncertain. While positive trends were noted, the highly inconsistent data across a limited number of studies indicate the data should be interpreted with caution. Furthermore, a substantial gap exists regarding underweight early adolescents. To address this, further high-quality trials, are needed particularly in low-income settings. Protocol registration: PROSPERO (CRD42024569008).

## Background

Early adolescence(ages 10–14 years) is a critical developmental period characterized by rapid growth, pubertal maturation, and substantial changes in body composition [1–7]. Nutritional requirements increase considerably during this stage, while dietary and physical activity behaviors established during adolescence can have lasting effects on health and development [8–10]. As a result, early adolescence represents an important opportunity to promote healthy growth and prevent both undernutrition and overweight.

Approximately 90% of the world’s adolescents (10–19 years) live in low- and middle-income countries (LMICs) [11], where undernutrition and overnutrition increasingly coexist as the “double burden malnutrition” (DBM). This phenomenon reflects a global transition, with the burden of overweight and obesity increasingly moving from high-income to resource-limited settings [12]. DBM is now predominantly concentrated in countries with much lower Gross Domestic Product per capita, particularly in the regions of South and East Asia and Sub-Saharan Africa [12–14]. Both forms of malnutrition are associated with adverse health, developmental, and long-term cardiometabolic outcomes [6, 15–22]. Importantly, early-life undernutrition may increase susceptibility to overweight and obesity later in life, illustrating the interconnected nature of DBM and the importance of addressing multiple forms of malnutrition simultaneously [22–24]. Beyond its physical health implications, body image dissatisfaction during adolescence is also associated with adverse psychological outcomes, including depression, anxiety, low self-esteem, and risky behaviors [25, 26].

Rapid urbanization and dietary transition in LMICs have contributed to increased consumption of energy-dense, nutrient-poor foods while reducing opportunities for physical activity [27–30]. Consequently, adolescents are increasingly exposed to obesogenic environments that increase the risk of unhealthy weight gain and cardiometabolic disorders [22–24, 31]. At the same time, chronic undernutrition may compromise physical fitness and healthy development, highlighting the complex interaction between nutrition, physical activity, and health during adolescence [30, 32–36].

Given the importance of modifiable lifestyle factors during adolescence, interventions that combine nutrition education and physical activity promotion have received growing attention [37–40]. Such education-based approaches may improve dietary behaviours, support healthy growth and development, and contribute to the prevention of both undernutrition and overweight. Evidence further suggests that interventions grounded in behavioural theory and supported by parental involvement are more likely to achieve favourable dietary and anthropometric outcomes [41, 42]. A recent review of community-based interventions in LMICs shows that most adolescents’ nutritional challenges are approached through single‑component strategies with a narrow focus, such as supplementation, nutrition education, physical activity education, or behavioral interventions focused on physical activity [38]. Yet, tackling multiple forms of malnutrition simultaneously across life courses is essential. In this context, double duty actions have been recommended as an efficient approach because they address several dimensions of malnutrition at once [37]. These actions are particularly important in countries undergoing nutrition transition and facing DBM.

Although a large body of evidence exists on nutrition and physical activity interventions among children and adolescents [38–40, 43–45], most studies have been conducted in high‑income countries and have focused on single outcome measures, such as body mass index (BMI). Existing reviews also indicate that interventions are frequently implemented as single-component strategies, whereas evidence regarding integrated education-based approaches among socioeconomically disadvantaged adolescents remains limited [45–47]. Evidence gaps are even more pronounced for socioeconomically disadvantaged adolescents, who face higher risks of multiple forms of malnutrition [48]. Furthermore, early adolescents are often overlooked as a distinct group despite their unique developmental needs and heightened vulnerability to multiple forms of malnutrition [49–51].

To address these evidence gaps, this systematic review specifically focuses on education‑based interventions combining nutrition education and physical activity promotion among socioeconomically disadvantaged early adolescents. Our review was designed to consider both underweight and overweight within the broader framework of double burden malnutrition. However, no eligible studies addressing underweight among disadvantaged adolescents were identified, and only overweight‑related interventions were found.

The primary objective of this review is to determine the effectiveness of combined physical activity and nutrition education interventions among socioeconomically disadvantaged early adolescents experiencing either underweight or overweight compared with single-component interventions or no intervention.

## Methods

### Protocol and registration

This systematic review was conducted according to the Preferred Reporting Items for Systematic Review and Meta-Analysis Protocols (PRISMA-P) 2020 statement (S1 File) [52]. The protocol for the review was registered in PROSPERO (CRD42024569008) before the initiation of the review. The review protocol can be accessed from the corresponding author.

### Information sources and search strategy

A comprehensive search was conducted in four databases: PubMed, Web of Science, Scopus and LILACS Plus collection. Both controlled terminology and free text terms were tailored to the specifications of each database (S1 Text). The search strings comprised three central topics: lifestyle modifications, preadolescent, and nutritional status (overweight or underweight) and low resource area.

The initial string was developed in PubMed and adapted to meet the requirements of the other databases. The primary and review articles’ references were cross-referenced to ensure the inclusion of all relevant articles. The search was last updated on June 18^th^ 2026.

### Selection process

The selection criteria were defined using population, intervention, comparison, outcome, and study design PICOS method [53].

#### Population.

Studies involving early adolescence (age 10–14 years) with DBM, encompassing both underweight and overweight, or either condition exclusively, were included. Studies were excluded if they only included children younger than 10 and older than 14. Underweight or overweight children on medical treatment, adolescents with chronic disease (e.g., diabetes, hypertension, metabolic syndrome), and neurological or neurodevelopmental disorders (including but not limited to Cerebral palsy, Attention Deficit/Hyperactivity Disorder, autism spectrum disorder) were also excluded.

Studies were eligible when the reported age range or mean age ± standard deviation indicated inclusion of participants within the 10–14 year age range, even if younger children were also included, because subgroup data for early adolescents were frequently unavailable.

#### Intervention.

Studies involving combined nutrition and physical activity (including usual and unstructured physical activity) education intervention promoting behavior change, healthy feeding practices, improved nutritional status, healthier body composition, and increased physical activity were included. Interventions including dietary supplementation, such as treatments for underweight, overweight, or obesity in early adolescents, were excluded. Interventions that included structured physical activity were also excluded, as evidence on their effect has already been established [54, 55].

#### Comparison.

Studies involving a single nutrition education intervention, or a single physical activity education and control groups (with no educational intervention) were included.

#### Study design and language.

All randomized controlled trials (RCTs), including cluster RCTs, published in all languages were included. All other types of intervention studies were excluded, including quasi-experimental designs, single-arm studies, observational studies, such as case-control and cohort studies, as well as editorials, and review articles.

In the initial phase, two reviewers (EV, RKA) independently screened all records. In the first screening phase, titles and abstracts were evaluated using PICOS criteria. In the second phase, full-text articles were assessed for eligibility (Fig 1). Any discrepancies between reviewers were resolved through discussion and consensus. Reasons for exclusion were systematically recorded at each stage of the screening process. There were no restrictions or filters applied.

**Fig 1:**
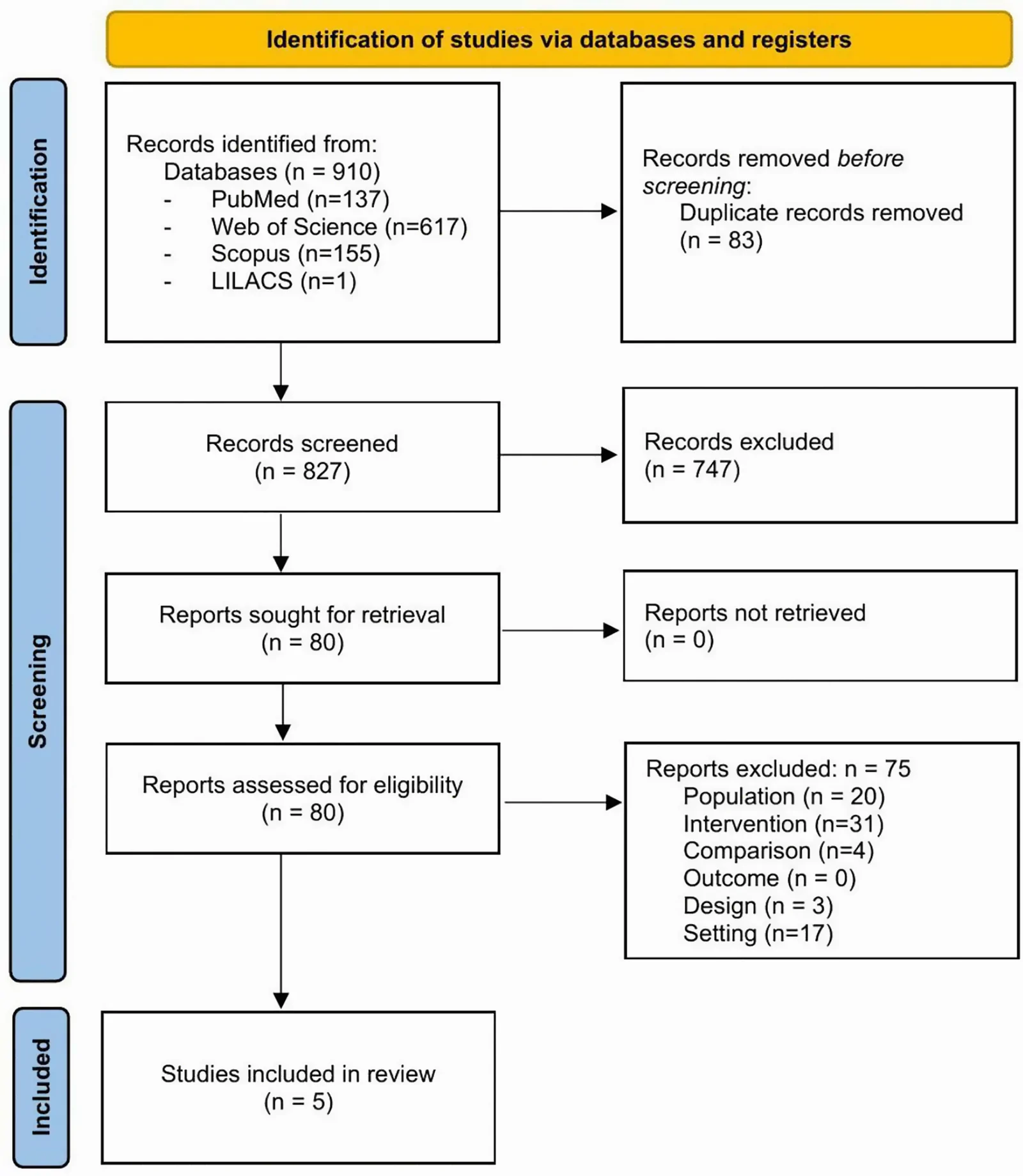
Prisma flow diagram of the articles identification and screening process.

### Methodological quality assessment

The risk of bias of the included RCTs was assessed using the revised Cochrane Risk of Bias Tool for Randomized Trials (ROB 2) by two reviewers (EV, RK) independently [56]. ROB 2 considers the five domains. The first domain addresses bias arising from the randomization process, which includes whether the sequence of allocation is random and concealed. The second domain considers bias due to deviations from the intended intervention, which includes whether (a) the participants are aware of assignments, (b) there are deviations from protocol, or (c) intention-to-treat analysis has been done. The third domain addresses bias due to missing outcome data, which includes whether data are missing for many participants and whether appropriate methods are used to handle missing data. The fourth domain rates bias in the measurement of outcome, which includes whether the outcome assessors are blinded, whether the measurement methods are appropriate, and whether knowledge of intervention affects measurement. The fifth domain addresses bias in the selection of reported results and incomplete outcome data, assessing whether the results align with pre-specified plans or are selectively reported. For each domain, the overall risk of bias was established: low risk of bias, high risk of bias, or some concern of bias. The risk of bias at the domain level was used to estimate the overall risk of bias: 1) low risk if all domains have a low risk of bias, 2) some concerns if one or more domains have some concerns but none of them high, and 3) high risk if one or more domains are labeled as high risk.

### Data extraction

The data were first extracted independently by one reviewer (RKA) and checked by a second reviewer (EV). The following information was extracted from the included studies: number of participants, adolescent’s age, pre- and post-intervention outcomes (mean, SD), sample size, type of underweight/overweight, setting, and country where the study was conducted, duration of intervention, family involvement in the intervention, type of applied electronic technology, and summary of the intervention description. The outcomes were categorized as: anthropometric measures (weight, height, BMI, waist circumference, hip circumference), body composition (Triceps Skinfold Thickness (TSF), Bioelectrical Impedance Analysis (BIA), physical activity, sedentary behavior, dietary behavior, nutritional knowledge, and biochemical blood profiles. For any missing or unclear information, study protocols and supplementary materials were explicitly checked, and unclear data were recorded as missing and during extraction. Both within-group changes and between-group differences were reported and tabulated for each outcome at the individual study level.

### Level of evidence

The Grading of Recommendations, Assessment, Development, and Evaluation (GRADE) approach was used to assess the quality of evidence [57]. Five domains were used to evaluate each outcome: study limitations (risk of bias), inconsistency of results, indirectness of evidence, imprecision, and publication bias. Each outcome underwent a detailed assessment of certainty, taking these domains into account.

Inconsistency was assessed by examining variability in the direction and magnitude of the intervention effects across studies and outcome domains. Precision was assessed by evaluating whether the available sample sizes were sufficient to support reliable conclusions regarding intervention effectiveness. Directness was evaluated by assessing the applicability of study populations, applied interventions, and outcome measures to the review question. The certainty of the evidence was assessed by one reviewer (RKA) and independently checked by a second reviewer (EV).

## Results

### Study selection

A total of 910 articles were retrieved from the four selected databases: 137 from PubMed, 155 from Scopus, 617 from Web of Science and 1 from LILACS. After removing 83 duplicates, 827 articles were screened on title and abstract, among which 747 were excluded. Eighty full texts were screened, 75 of which were excluded. Finally, 5 studies were included in the systematic review. The complete flow of study selection is presented in Fig 1.

### Methodological quality

Choo et al. [58] was rated as having a low risk of bias, while the other selected studies were assessed as having some concern, each relating to a different bias domain. Risk of bias assessment. shows that there were no consistent methodological errors present across the included studies Fig 2.

**Fig 2:**
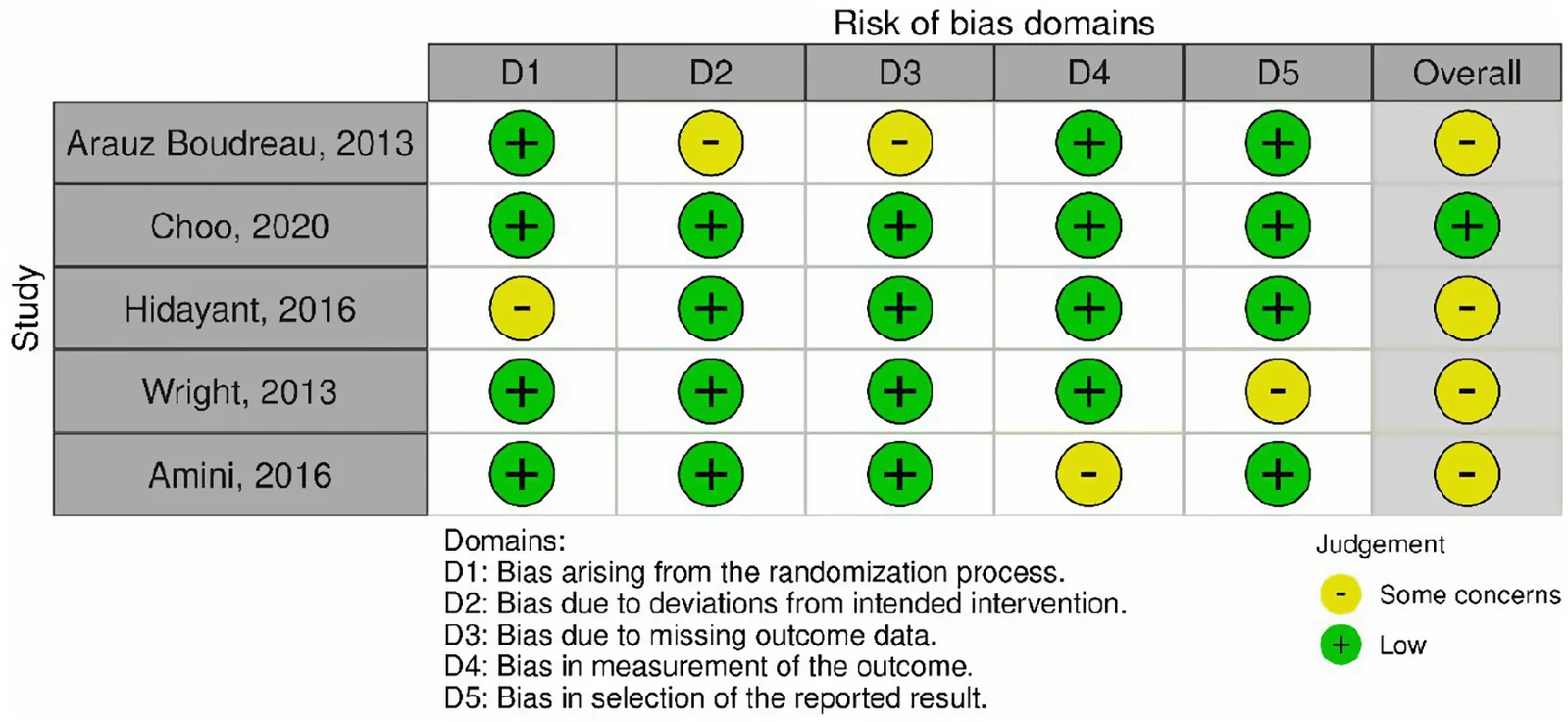
Risk of bias assessment.

### Participants

Overall, a total of 960 early adolescents participated in the included studies. The sample size ranged from 41 [59] to 334 [60], with the mean age of the participants being 10.6 ± 1.4 years. All included children were overweight or were having obesity with no studies including underweight children. Three studies were conducted in high-income countries (South Korea, the United States, respectively), focusing specifically on minority or socio-economically vulnerable children within school settings, community health centers, and in public welfare settings [58, 59, 61]. Two studies were conducted in a school setting in lower-middle-income countries (Indonesia & Iran, respectively) [60, 62]. None of the studies were conducted in low-income countries. The complete description of individual study characteristics is presented in Table 1.

**Table 1:** Description of the intervention with the outcomes.

Authors (year)	Intervention groups	Intervention volume	Category of the outcome measure	Outcome measures	Within group comparisons		Between group comparison	RoB2 score
					IG	CG		
Amini 2016	**IG:** Nutrition education, PA education and lifestyle education **CG:** No intervention	For NE -12 weeks -1 session per week -15 to 45 minutes per Session Total- 12 sessions For PE -12 weeks - 45-2hr a week	**Anthropometry**	BMI Z-score	*	*	IG > CG	Some concern
				Hip circumference	*	*	IG > CG	
				Waist circumference	*	*	IG > CG	
				triceps skin-fold thickness	ns	*	IG > CG	
			**Physical activity**	Duration of PA of MET 3 to 6 /day (min)	*	*	IG < CG	
				Duration of PA of> MET7/day (min)	*	*	IG > CG	
			**Sedentary Behaviors**	Duration of TV viewing/day (hr.)	ns	ns	IG = CG	
				Duration of working with computer/day (hr.)	ns	*	IG > CG	
			**Energy and Dietary Intake**	Energy,	*	ns	IG < CG	
				Protein	*	ns	IG<CG	
				Fat	*	ns	IG<CG	
				carbohydrate	ns	ns	IG<CG	
				% energy fat	*	ns	IG<CG	
				% energy carbohydrate	*	ns	IG<CG	
Arauz Boudreau 2013	**IG**- Healthy behaviors surrounding nutrition, activity, and stress management. Physical activity education **CG**- No intervention wait list	For PA &NE -5 weeks -1 session per week -Total Ongoing coaching Over 6months	**Anthropometry**	BMI z score	ns	ns	IG = CG	Some concern
			**Blood and metabolic** **markers**	(Lipids, Triglycerides, LDL, HDL, Glucose, Insulin, HbA1C)	ns	ns	IG = CG	
			**Physical activity**	MVPA	ns	ns	IG = CG	
Choo 2020	IG-multi-level strategies (Child-level education based on 5-2-1-0 principles, parent-level counseling/home visits, and center-level policy changes. On healthy eating principles of physical activity CG- No intervention	For PA &NE -12 weeks -6 sessions for NE -6 sessions for PA	**Anthropometry**	BMI for z score	ns	ns	IG = CG	Low risk
				Obesity status	ns	ns	IG=CG	
			**Nutritional**	vegetable intake	*	*	IG > CG	
				Healthy eating behaviors	*	*	IG > CG	
				family mealtime meal intake	*	ns	IG > CG	
			**Physical activity**	moderate physical activity	*	ns	IG > CG	
			**Sedentary behavior**	screen time	ns	ns	IG = CG	
Hidayanty, 2016	**IG**- nutrition education, physical activity and healthy eating **CG**- received leaflet based on nutrition information	For PA &NE -12 weeks -1 session per week -75 minutes per session Total 12 sessions	**Anthropometry**	BMI z-score	*	ns	IG > CG	Some concern
				Waist circumference	*	ns	IG>CG	
			**Nutrition**	snacking habits	*	ns	IG > CG	
			**Sedentary behavior**	sedentary activity	ns	ns	IG = CG	
			**Psychosocial**	Self-efficacy for reducing snacking	*	ns	IG > CG	
				Self-efficacy for reducing sedentary behaviors	*	*	IG > CG	
Wright 2013	**IG**- Lifestyle intervention program, Kids N Fitness. Physical activity and nutrition education. **CG**- No intervention participated in standard physical activity	For PA and NE -6-week -1 session per week - 90-min sessions per week - 45 minutes for PA -45 minutes for NE	**Anthropometry**	BMI z score	*	ns	IG > CG§	Some concern
				waist circumference	ns	ns	IG = CG	
			**Physical activity**	Physical activity 60 min/day	*	ns	IG>CG§	
				Daily physical education class attendance	*	ns	IG > CG§	
			**Sedentary behavior**	TV viewing	*	ns	IG > CG♂	
				Computer/video game	ns	ns	IG = CG	

**Abbreviations**: HDL, high density lipoprotein, LDL, low density lipoprotein, BMI Z score, body mass index z score, AST, aspartate aminotransferase, and ALT, alanine aminotransferase**:** Intervention Group, **CG:** Control Group / Comparison Group, **RoB2:** Risk of Bias in Randomized Trials, **BMI:** Body Mass Index, **PA:** Physical Activity, **MET:** Metabolic Equivalent of Task, *: Statistically significant change/effect (p < 0.05), **ns:** Non-significant change/effect (p > 0.05), IG = CG No statistically significant difference was found between the intervention and control groups. IG > CG The intervention group achieved a statistically significant, superior health outcome compared to the control group. IG < CG The control group experienced a statistically significant, greater change than the intervention group. § girls only. ♂ boys only

#### Intervention characteristics Nutrition and physical activity education.

Various combined nutrition and physical activity education interventions were implemented to improve weight status, enhance physical activity levels, and promote healthier dietary habits among overweight and adolescents with obesity. The overall intervention duration ranged from six [61] to 18 [60] weeks. Education sessions were held weekly [58–62], with the total session delivery time varying from one and half months [58, 59] to 3 months [60–62]. Education sessions occurred at school during school time [60, 62] or after school hours [61], at the health center [59], or in community child centers of public welfare [58], once per week, lasting between 75 [62] to 90 [61] minutes.

**Nutrition education sessions** focused mainly on healthy eating habits, including portion control, and reducing snacking habits, increasing knowledge about food groups, energy, and quality of the foods (such as increasing fruit and vegetable consumption, the dangers of liquid calories), but also the need for calorie restriction by limiting carbohydrate intake and reducing total saturated fat and cholesterol. In addition, nutrition education focused on behavioral modification sessions related to nutrition quality, the relation between healthy eating and health outcomes, self-efficacy enhancement, understanding nutrition fact labels, and healthy food shopping at the school site.

**Physical activity education interventions** mainly focused on increasing physical activity and reducing sedentary behaviors [62]. In addition, physical activity components included instruction in the importance of warming up, stretching, and maintaining adequate hydration during exercise. The lessons were reinforced at home with the use of journals, assignments, and giveaway items to promote physical activity at home, e.g., by giving balls for children and pedometers for caregivers [59]. Moreover, the healthy activity sessions included information on principles of desirable physical activity and health outcomes, safe activity practice, and disadvantages of sedentary behaviors [58]. Additionally, educational sessions included information and examples of age-appropriate physical activities such as walking, running, dancing, and informal games; however, structured physical activity sessions were not delivered as part of the intervention. The physical activity education was given with a duration ranging from 45 [61] min to two hours [60] a week in addition to regular sport sessions at school [60, 62] and taught by physical education teachers.

In all of the interventions, parents were actively involved in which monthly education sessions were given from 45 [61] to 90 [60] minutes approximately over 4 months. The sessions for parents focused on educating healthier cooking methods, promoting physical activity, and educating them about childhood overweight and obesity. Additional support for parents was provided through providing weekly nutrition education leaflets over 12 weeks and structured parenting guidance [62]. In addition, parenting skills training includes goal setting, self-monitoring, reinforcement strategies, and fostering a supportive home environment. It was delivered through a combination of one group teaching home visits, three telephone counseling calls, and 12 text messages to reinforce key messages [58, 59].

Two of the interventions were theory-based [58, 62]. One of them utilized the Social Cognitive Theory (SCT), which is a behavior change theory that explains how individuals learn and change behavior through the interaction of personal factors, environmental influences, and behav]. A key concept is observational learning, where people learn by observing others, with specific strategies for managing behavioral changes related to goal setting, dealing with stress, and building self-esteem [62,63]. Another was guided by cognitive learning theory, a learning theory that focuses on mental processes such as memory, problem-solving, and information processing in learning [64]. Learning is considered an active process of acquiring and organizing knowledge [58].

In some of the studies [58, 60, 61], the centers and schools were also involved as part of the program, in which the schools were asked to make healthy foods more attractive, to stop selling high-calorie food and drinks, minimize the use of oils, and to serve more vegetables, legumes, and whole wheat bread. Along with encouraging the students to increase moderate physical activity and reduce sedentary behaviors. Moreover, the researchers educated the faculty members to be aware of obesity prevention and its related positive behaviors. Teachers received weekly nutrition training aligned with children’s curriculum [58, 60]. Despite some variations in setting and content delivery, the overall modality was similar, emphasizing interactive and group-based learning.

#### Controls.

Across the included trials, the control groups received interventions with leaflets on evidence-based nutrition information [62], a standard physical activity program given by the respective schools [61], or usual care being provided by the center, which did not include any nutrition and physical activity education [58]. In one study, the control group did not receive any intervention [60]. However, in one of these trials, participants in the control group were placed on a waitlist and received the intervention 6 months later, after the intervention group [59].

### Outcome measures

All of the interventions assessed anthropometric outcomes such as BMI [58–62], weight, height [59], and waist circumference [59–62], hip circumference, and skinfold thickness [60] to evaluate physical health changes. In addition, in one of the study incorporated blood and biochemical markers variables such as total cholesterol, low-density lipoprotein, high-density lipoprotein, very low-density lipoprotein, liver enzymes (including aspartate aminotransferase, alanine aminotransferase) [59], or behavioral outcomes such as dietary intake, snacking frequency [62], and physical activity levels [58–61] assessed through self-report [58, 60, 61], parent report, or using accelerometers [59]. Importantly, family involvement was consistent in all interventions. The family nutrition and physical activity scale was used in one study to assess parenting behaviors specifically related to obesity [58].

### Effect of intervention on the outcomes

Table 1 provides an overview of the intervention effects at individual study level, grouped per outcome measures.

Five studies assessed intervention effects on anthropometric outcomes, including BMI z- score, [58–62] waist circumference [60–62], hip circumference [60], and triceps skin-fold thickness [60]. Three studies demonstrated significantly greater reduction in BMI z-score among participants in the intervention group compared with the control group [60–62], whereas two studies reported no statistically significant difference in BMI z-scores between groups [58, 59]. A significant reduction in waist circumference [62] and hip circumference [60] was also observed among intervention participants. One of the interventions also demonstrated a protective effect, successfully preventing significant increase in triceps skinfold thickness and waist circumference [60].

Across three studies that assessed nutrition related outcomes [58, 60, 62], three studies showed significant improvements in general nutrition knowledge, healthy lifestyle behaviours specifically related to increase in vegetable intake, and family mealtime habits [58]. Furthermore, among overweight adolescents, on of the combined education interventions successfully reduced snacking frequency and raised snacking self-efficacy [62]. However, Amini et al. reported significant increases in total energy, fat and carbohydrate intake, in no sugar sweetened beverage, and fast food consumption were observed within the intervention group [60].

Four studies assessed physical activity using validated questionnaires and accelerometers [58–61]. Overall, findings suggest that combined nutrition and physical activity education interventions may improve physical activity behavior, although results were inconsistent across studies. Three studies reported favorable effects on the intervention, including increases in daily physical activity [61],vigorous physical activity [58, 60], and physical education class attendance [58, 61] partiuclarly among girls. In one study moderate physical activity increased in the control group, while the education group showed a significant increase in vigorous physical activity [60]. In another study significant increases in 60 minutes of moderate physical activity were observed [58]. In contrast, in one studie no significant differences were noted in physical activity levels between the intervention and control groups, with physical activity levels declining in both groups [59].

Four studies evaluated sedentary behavior [58, 60–62], primarily through measures of television viewing time and computer use. Overall, results were mixed. Two studies reported significant difference in sedentary behaviors or television viewing time between the intervention and control groups [60, 61], while two did not [58, 62]. The intervention resulted in significant improvement by decreasing TV viewing among boys [61]. In contrast, computer use increased in both groups; however, the increase was much smaller in the intervention group compared to the control [60].

### Level of evidence

According to the GRADE methodology, all studies contributing to the outcomes were randomized controlled trials (2 RCT and 3 cRCT); therefore, the certainty of evidence started high. No downgrading was applied for study limitations because the included studies demonstrated low to acceptable risk of bias. The level of evidence was downgraded by two levels due to substantial inconsistency in results. The certainty of evidence was downgraded by one level for imprecision because the evidence was based on a limited number of studies with relatively small sample sizes and heterogeneous outcome measures, resulting in uncertainty regarding the magnitude of intervention effects.The final grading level was very low certainty for all outcomes combined. New methodologically sound trials may likely change the currently available conclusions (Table 2).

**Table 2:** Quality of evidence appraised using the Grading of Recommendations, Assessment, Development and Evaluation (GRADE)-method.

Studies	Study limitation	Inconsistency	Indirectness	Imprecision	Quality of evidence (Grade)
All together [58**–**62]	=	↓↓	=	↓	⨁◯◯◯

Explanation: ↓ GRADE score downgraded by one point; = No impact on GRADE score; there was some uncertainty about downgrading the evidence because of indirectness, as recommended by the GRADE guidelines.; ⊕ Point on final GRADE score awarded; ⊖ Point on final GRADE score not awarded and suggested representations of the quality of evidence; Symbol ⨁⨁⨁⨁ = high ⨁⨁⨁◯ = moderate ⨁⨁◯◯ = low, and ⨁◯◯◯ = very low.

## Discussion

This systematic review evaluated the effectiveness of combined nutrition and physical activity educational interventions on anthropometric outcomes, dietary habits, physical activity and sedentary behaviors. The available evidence suggests that combined physical activity and nutrition education interventions may improve anthropometric indicators, dietary behaviors, physical activity levels and sedentary behaviors among overweight and obese early adolescents. However, the certainty of evidence was graded as very low, indicating that confidence in the estimated effects is limited and that the true effect may differ substantially from the observed findings. Importantly, none of the included studies investigated lifestyle interventions among underweight early adolescents, highlighting a critical gap in the current evidence base for this population.

Evidence for the effect of combined education interventions on BMI z-scores was inconsistent, as some studies reported significant improvements whereas others found no significant effects [58–62]. Similar inconsistencies have been reported in previous systematic reviews, with some demonstrating reductions in BMI, BMI z-score, and body weight [43, 44, 47, 65–67], whereas others found little or no measurable effect [39, 49, 68]. These mixed findings suggest that the effectiveness of interventions may be influenced by differences in intervention content, duration, implementation setting, participant characteristics and outcome assessment methods. Despite this heterogeneity, some common factors may contribute to the positive effects observed across studies. Overweight and obesity in both high-income countries and LMICs are driven by similar underlying factors, including sedentary lifestyles and increased consumption of energy-dense, ultra-processed foods [69]. Consequently, interventions targeting these shared behavioural risk factors may yeild comparable benefits across settings. However, important contextual differences remain. Socioeconomically disadvantaged populations may face unique barriers to healthy eating and physical activity and may adopt different coping strategies during food insecurity, which could hamper intervention effectiveness.

The limited consistency across interventions in terms of their components may explain the lacking intervention effects. Furthermore, most included interventions were conducted in high-income countries and paid limited attention to equity-related factors. These contextual differences may partly explain the variability in intervention effectiveness and limit the generalisability of the findings to disadvantaged populations in LMICs.

The findings of this review suggest improvements in nutrition-related outcomes, including nutrition knowledge, vegetable intake, family mealtime habits, overall nutrition scores, and reductions in snacking behaviors [58, 62]. These finidngs are consistent with evidence from other studies in socioeconomcially disadvantaged populations reporting meaningful improvements in dietary intake, healthier fat consumption and posititve dietary behavior change [70]. Furthermore, these findings indicate that education-based interventions may improve dietary behaviours among early adolescents despite the socioeconomic challenges faced by the target populations. However, not all dietary outcomes were favorable. One study demonstrated that energy and fat intake as well as total energy derived from fat and carbohydrate increased significantly over the course of the intervention group. These findings appear inconsistent with the anthropometric outcomes, which showed reductions in adiposity slower increases in adiposity among intervention participants compared with controls [60]. This discrepancy suggests that changes in dietary composition alone may not fully explain improvements in anthropometric outcomes. Instead, intervention effects may reflect the combined influence of multiple behavioural changes, including increased physical activity, reduced sedentary behaviour, and broader lifestyle modifications.

Across four studies assessing physical activity outcomes following combined interventions, three reported significant increase in activity levels [58, 60, 61]. These findings align with evidence showing that combined exercise and behavioral strategies can improve physical activity intensity, daily steps, leisure activity scores and motivation to be active [38, 44]. School based interventions using simple environmental modifications or unstructured free play also increased time spent in moderate to vigorous physical activity [71]. Notably, successful interventions did not require large financial investments, making them particularly suitable for adolescents in low socioeconomic settings [72]. Among adolescents, interventions delivered in primary care settings were notably effective, especially those incorporating tailored advice or brief counseling [73]. In contrast two studies, however, reported non-significant changes in physical activity [59].

Regarding sedentary behaviors, one intervention reported a significant reduction in television viewing among boys [61] and improved self-efficacy related to sedentary behaviors [62]. However, other studies found no significant changes in sedentary behaviors or even reported increased television viewing in the intervention group [58, 60]. These findings are consistent with previous evidence showing that reducing sedentary behaviours is challenging among overweight adolescents and that intervention effects on television viewing are often limited [74, 75]. intervention resulted in a significant reduction in television viewing among boys and improved self‑efficacy related to sedentary behaviors. This finding aligns with evidence consistently showing that males tend to have higher activity levels than females, which may influence patterns of sedentary behavior [76, 77]. However, Gortmaker reported that reductions in television viewing predicted changes in obesity among girls, but not boys [78]. Among girls, each hour of reduced television viewing was associated with lower obesity prevalence. These sex‑specific differences highlight the need for interventions that are tailored to both boys and girls, particularly in low‑resource settings where targeted strategies may be essential for effectiveness.

Importantly, longer television viewing time has been associated with higher increased BMI and obesity risk [79], highlighting the continued importance of targeting sedentary behaviour in obesity prevention interventions.

Several interventions included behavioral theories such as Social Cognitive Theory and Cognitive Learning Theory, which may have contributed to improvements in BMI, physical activity and sedentary behaviors [58, 62]. This is consistent with previsous evidence suggesting that theory-based nutrition education interventions produce more sustained behavioral change [41].

Across the included studies, interventions incorporating both nutrition and physical activity education, particularly those involving parents, appeared to be associated with more favourable outcomes than single-component interventions or no intervention [58–62]. These findings align with previous reviews demonstrating the benefits of multicomponent, family-based approaches for improving dietary behaviours and reducing BMI among children and adolescents [42, 80]. Moreover, our results align with previous systematic reviews showing that long-term multicomponent interventions with parental involvement demonstrated better effect in reducing the BMI of children and adolescents with overweight [81–83].

Despite their potential, lifestyle interventions remain underutilized among disadvantaged populations, particularly in LMICs [84]. Moveover, interventions in sub-Sarahan African regions continue to focus predominantly on undernutrition, while obesity prevention is prioritized in wealthier countries, highlighting important gap in addressing the double burden of malnutrition [85]. Further research is therefore still needed to evaluate the effectiveness of combined intervention among socioeconomically disadvantaged early adolescents in low-income settings [48].

### Strengths and limitations

Although the number of studies included was relatively small, the review features a robust research search and a systematic approach to synthesize evidence on multicomponent lifestyle interventions, comprising both nutrition and physical activity education, compared to education-only interventions, without restricting the publication date. The absence of studies from low-income countries may limit the generalizability and external validity of the findings. Furthermore, no information was retrieved on undernourished preadolescents, clearly confirming a persistent gap in the current literature.

### Clinical implications and recommendations

Currently, there is a high prevalence of undernutrition and increasing obesity in LMICS, especially in the sub-Saharan African region, among children and adolescents [86]. Unfortunately, achieving the Sustainable Development Goals of ending hunger and all forms of malnutrition by 2030 will be difficult in this region [87]. Meeting SDG 2 (zero hunger) requires the implementation of targeted that address future generations, starting from infancy through early adolescents, as this specific period offers a second window of opportunity for development.

Given our current findings, interventions should be considered in school settings within low- resource areas, including in low-income countries. Adolescents from LMICs face food insecurity, a known cause of undernutrition and stunting, which now simultaneously linked to overweight and obesity due to the affordability of high-energy processed foods with low diversity [88]. The absence of research from LMICs targeting early adolescents underscores the urgent need for additional studies. Specifically, investigating the effect of combined interventions that simultaneously address the prevention of overweight and obesity while reducing the risk of undernutrition is imperative to understand the effects among early adolescents with DBM.

While education interventions are valuable in improving BMI, further research is needed to determine how they can be optimized or combined with other strategies such as dietary modification, physical activity, and family engagement. Perhaps even more aspects of development in the future including cognitive and executive functioning, motor skills. To generate a high level of evidence, well-designed, theory-driven (e.g., SCT) and rigorous evaluation methods are essential. In general, this systematic review directed that further primary and intervention studies are required to evaluate the effectiveness of the combined intervention in low-income settings among early adolescents.

## Conclusion

This systematic review found that combined interventions may improve BMI among overweight and obese early adolescents. However, the certainty of evidence was very low, and the findings should therefore be interpreted with caution. Further high-quality, well-reported trials are needed to strengthen the evidence base. Althugh this review focused on socio-economically disadvantaged populations, there remains a critical lack of evidence from low-income countries. Policymakers and researchers should prioritize early adolescents in these settings, where the double burden of undernutrition and overnutrition is increasingly prevalent. Future interventions should be tailored to the unique challenges of low-income contexts and evaluated using rigorous study designs to ensure effectiveness, equity, and sustainability in addressing adolescent health.

## Supporting information

S1 File PRISMA 2020 Checklist. Prisma 2020 checklist for reporting this systematic review.
(DOCX)

S1 Text Search strategy for all databases. The complete electronic search strategies used for all data bases included in this systematic review.
(DOCX)

## Abbrevations:

LMICs: Low- and middle-income countries
DBM: Double burden malnutrition
BMI: Body mass index
PRISMA-P: Preferred Reporting Items for Systematic Review and Meta-Analysis Protocols
RCTs: Randomized controlled trials
ROB 2: Cochrane Risk of Bias Tool for Randomized Trials
TSF: Triceps Skinfold Thickness
BIA: Bioelectrical Impedance Analysis
SPSS: Statistical Package for Social Sciences
GRADE: Grading of Recommendations, Assessment, Development and Evaluation
SDGs: Sustainable Development Goals
SCT: Social Cognitive Theory
