# Supplementary material for: Combined nutrition and physical activity education for early adolescents with malnutrition: A systematic review of overweight interventions and the undernutrition evidence gap
Source: PLOS Glob Public Health. 2026 Sep 29;6(9):e0007280. doi: 10.1371/journal.pgph.0007280 (PMC13623121; doi:10.1371/journal.pgph.0007280)
Supplement: Supplementary file 2 [file pgph.0007280.s002.docx]

# **Search strings for PubMed, Web of Science and Scopus**

**PubMed**: (("life style"[MeSH Terms] OR "life style*"[Text Word] OR lifestyle[Text Word]) AND ("modification"[Text Word] OR intervention[Text Word] OR strategy[Text Word] OR strategies[Text Word] OR program[Text Word] OR programs[Text Word])) OR "Health education"[MeSH Terms] OR (("physical activity education"[Text Word]) AND (((nutrition[Text Word] OR eating[Text Word]) AND (education[Text Word] OR "modification"[Text Word] OR "intervention"[Text Word] OR strategy[Text Word] OR strategies[Text Word] OR program[Text Word] OR programs[Text Word])) OR "nutrition education"[Text Word])) AND (("adolescent" [MeSH Terms] OR adolescen*[Text Word] OR "child"[MeSH Terms] OR children[Text Word] OR child*[Text Word] OR "early adolescent"[Text Word])) AND (("malnutrition"[MeSH Terms] OR malnutrition[Text Word] OR "nutritional deficiency"[Text Word] OR "Malnourish*"[Text Word] OR "Undernourished"[Text Word] OR "Undernutrition"[Text Word] OR "under-nutrition"[Text Word] OR "thinness"[MeSH Terms] OR leanness[Text Word] OR underweight[Text Word] OR "cachexia"[MeSH Terms] OR wasting[Text Word] OR "growth disorders"[MeSH Terms] OR stunting[Text Word] OR "Overweight"[Mesh Terms] OR "Obesity"[Mesh Terms] OR "over-weight"[Text Word] OR "double burden malnutrition"[Text word])) AND ("low and middle income country"[Text Word] OR "LMIC"[Text Word] OR "low-resourced area"[Text Word] OR "poverty"[Text Word] OR "disadvantaged"[Text Word] OR "poverty areas"[MeSH Terms] OR "vulnerable populations"[MeSH Terms]) AND ("Controlled Clinical Trial"[Publication Type] OR "controlled clinical trial"[Text Word] OR "Randomized Controlled Trial"[Text Word]) N= 137, June 18^th^ 2026

**Web of Science**

TS=((“adolescent” OR “adolescen*” OR “early adolescent”) AND (“malnutrition” OR “nutritional deficiency” OR “Malnourish*” OR “Undernourish*” OR “Undernutrition” OR “under-nutrition” OR “thinness” OR “leanness” OR “underweight” OR “cachexia” OR “wasting” OR “growth disorders” OR “stunting” OR “Overweight” OR “Obesity” OR “over-weight” OR “double burden malnutrition”) AND (((“life style” OR “life styles” OR “lifestyle”) AND (“modification” OR “intervention” OR “program” OR “programs” OR “strategy” OR “strategies”)) OR ("Health education") OR (“physical activity education” AND (("nutrition" Or “eating”) AND (“intervention” OR “education” OR “modification” OR “program” OR “programs” OR “strategy” OR “strategies”)))) AND ("low and middle income country" OR "LMIC" OR "low-resourced area" OR "poverty" OR "disadvantaged" OR "poverty areas" OR "vulnerable populations") AND (“randomized controlled trial” OR “controlled clinical trial” OR “randomized controlled trials” OR “controlled clinical trials” OR RCT OR CCT))

N=617, June 18^th^ 2026

**Scopus**

((INDEXTERMS( "life style" ) OR TITLE-ABS-KEY( "life style*" ) OR TITLE-ABS-KEY( "lifestyle" )) AND (TITLE-ABS-KEY( "modification" ) OR TITLE-ABS-KEY( "intervention" ) OR TITLE-ABS-KEY( "strategy" ) OR TITLE-ABS-KEY( "strategies" ) OR TITLE-ABS-KEY( "program" ) OR TITLE-ABS-KEY( "programs" ))) OR INDEXTERMS( "health education" ) OR ((TITLE-ABS-KEY( "physical activity education" )) AND (((TITLE-ABS-KEY( "nutrition" ) OR TITLE-ABS-KEY( "eating" )) AND (TITLE-ABS-KEY( "education" ) OR TITLE-ABS-KEY( "modification" ) OR TITLE-ABS-KEY( "intervention" ) OR TITLE-ABS-KEY( "strategy" ) OR TITLE-ABS-KEY( "strategies" ) OR TITLE-ABS-KEY( "program" ) OR TITLE-ABS-KEY( "programs" ) )) OR TITLE-ABS-KEY( "nutrition education" ))) AND ((INDEXTERMS( "adolescent" ) OR TITLE-ABS-KEY( "adolescen*" ) OR INDEXTERMS( "child" ) OR TITLE-ABS-KEY( "children" ) OR TITLE-ABS-KEY( "child*" ) OR TITLE-ABS-KEY( "early adolescent" ))) AND ((INDEXTERMS( "malnutrition" ) OR TITLE-ABS-KEY( "malnutrition" ) OR TITLE-ABS-KEY( "nutritional deficiency" ) OR TITLE-ABS-KEY( "malnourish*" ) OR TITLE-ABS-KEY( "undernourished" ) OR TITLE-ABS-KEY( "undernutrition" ) OR TITLE-ABS-KEY( "under-nutrition" ) OR TITLE-ABS-KEY( "thinness" ) OR TITLE-ABS-KEY( "leanness" ) OR TITLE-ABS-KEY( "underweight" ) OR INDEXTERMS( "cachexia" ) OR TITLE-ABS-KEY( "wasting" ) OR INDEXTERMS( "growth disorders" ) OR TITLE-ABS-KEY( "stunting" ) OR INDEXTERMS( "overweight" ) OR INDEXTERMS( "obesity" ) OR TITLE-ABS-KEY( "over-weight" ) OR TITLE-ABS-KEY( "double burden malnutrition" ))) AND (TITLE-ABS-KEY( "low and middle income country" ) OR TITLE-ABS-KEY( "LMIC" ) OR TITLE-ABS-KEY( "low-resourced area" ) OR TITLE-ABS-KEY( "poverty" ) OR TITLE-ABS-KEY( "disadvantaged" ) OR INDEXTERMS( "poverty areas" ) OR  INDEXTERMS( "vulnerable populations" )) AND ( TITLE-ABS-KEY( "controlled clinical trial" ) OR TITLE-ABS-KEY( "randomized controlled trial" )) N=155, June 18th 2026

**LILACS Plus collection**

((mh:"life style" OR tw:"life style*" OR tw:"lifestyle") AND (tw:"modification" OR tw:"intervention" OR tw:"strategy" OR tw:"strategies" OR tw:"program" OR tw:"programs")) OR mh:"health education" OR ((tw:"physical activity education") AND (((tw:"nutrition" OR tw:"eating") AND (tw:"education" OR tw:"modification" OR tw:"intervention" OR tw:"strategy" OR tw:"strategies" OR tw:"program" OR tw:"programs")) OR tw:"nutrition education")) AND ((mh:"adolescent" OR tw:"adolescen*" OR mh:"child" OR tw:"children" OR tw:"child*" OR tw:"early adolescent")) AND ((mh:"malnutrition" OR tw:"malnutrition" OR tw:"nutritional deficiency" OR tw:"malnourish*" OR tw:"undernourished" OR tw:"undernutrition" OR tw:"under-nutrition" OR tw:"thinness" OR tw:"leanness" OR tw:"underweight" OR mh:"cachexia" OR tw:"wasting" OR mh:"growth disorders" OR tw:"stunting" OR mh:"overweight" OR mh:"obesity" OR tw:"over-weight" OR tw:"double burden malnutrition")) N=1, June 18^th^ 2026
